# Universal Child Mental Health Screening for Parents: a Systematic Review of the Evidence

**DOI:** 10.1007/s11121-024-01693-8

**Published:** 2024-06-16

**Authors:** Shona K. Brinley, Lucy A. Tully, Talia Carl, Rebecca K. McLean, Caitlin S. M. Cowan, David J. Hawes, Mark R. Dadds, Jaimie C. Northam

**Affiliations:** 1 The School of Psychology, The Faculty of Science, The University of Sydney, Sydney, NSW Australia; 2 The School of Psychology, The Faculty of Science, The University of New South Wales, Sydney, NSW Australia

**Keywords:** Child mental health, Parent-report, Universal screening, Effectiveness, Acceptability

## Abstract

Childhood represents a critical window for the emergence and treatment of mental health disorders, yet many are not being identified, or are identified too late to receive adequate intervention. This systematic review (Prospero registration: CRD42022299560) aimed to determine the effectiveness and acceptability of parent reported universal mental health screening (UMHS) to improve the early identification of children at-risk of mental health difficulties, and to identify barriers and enablers that may influence parental engagement. Six databases were searched in February 2022 for peer-reviewed, primary research. Studies conducted in targeted populations, evaluating psychometric properties, or focused on screening non-psychological problems were excluded. Ten studies examined parent reported (*n* = 3,464 parents) UMHS for children from birth to 18 years, suggesting an overall scarcity of research. Findings are presented in a table of study characteristics and a narrative summary of acceptability, effectiveness, barriers, and enablers. Quantitative findings indicated that parents generally support and accept UMHS. Research assessing effectiveness was limited, although two studies indicated increased referrals and referral adherence following positive screens. Confidentiality and stigma were commonly identified barriers. Quality assessment using the Mixed Methods Appraisal Tool indicated that studies varied in quality, meeting four to seven of the seven quality criteria. Understanding and addressing parent attitudes to UMHS across settings is necessary for the successful implementation of screening and improvement of child mental health outcomes. More high-quality research studies, including randomized controlled trials are therefore needed to examine the acceptability and effectiveness of UMHS for parents and their children.

Childhood is a critical period for the onset of mental health (MH) disorders (Colizzi et al., 2020). Half of all MH disorders emerge before the age of 14 years (Kessler et al., 2005) and three quarters by the age of 25 (McGorry & Mei, 2018). Of those children and youth with MH problems, many do not receive the necessary support. As a result, access to intervention remains low, and despite evidence that early intervention is key to improving long-term MH outcomes, interventions are often received only by those with severe disorders (Costello et al., 2014; Hiscock et al., 2020; Lawrence et al., 2015). For those who do receive help, it is often delayed and insufficient in duration or frequency (Lawrence et al., 2015; Sawyer et al., 2018a). Thus, improved efforts are needed to identify children at risk of MH disorders and provide effective intervention to alter the long-term trajectory of mental illness, and ultimately reduce the high and stable prevalence of childhood MH disorders (Sawyer et al., 2018b). Universal mental health screening (UMHS) offers a mechanism for early identification of children with emerging MH problems and timely referral to appropriate interventions. Parents are well-placed to conduct this screening as they are most familiar with their child’s needs and are the gatekeepers for their access to MH services and supports (Reardon et al., 2017).

UMHS involves a population-based approach to the implementation of validated screening measures to identify individuals who are at risk of MH problems. When implemented at key developmental timepoints, UMHS enables the proactive identification of children at-risk of MH problems identified early in a child’s development or in the trajectory of the MH disorder, and the subsequent provision of targeted support or early interventions to reduce or prevent the escalation of symptoms (Humphrey & Wigelsworth, 2016). Across a broad spectrum of MH domains, including internalizing, externalizing, and attentional disorders, UMHS can provide a contrast to other arbitrary, often inefficient approaches such as the ‘refer-test-place’ model, where individual children are referred to a professional for assessment and treatment (Dowdy et al., 2010), or the ‘wait-to-fail' model, where children at risk of MH problems are identified through mechanisms such as school absenteeism or office discipline referrals (Glover & Albers, 2007). In these approaches, identification often occurs only when MH problems are severe and entrenched, and therefore require more intense and costly interventions, which are often delivered too late, tend to result in under-referral, and disproportionally overlook vulnerable children (Humphrey & Wigelsworth, 2016). The United States Preventative Services Task Force advocates for UMHS for some conditions, such as major depressive disorder in adolescents, however, a notable gap exists in the research pertaining to the potential benefits and harms of screening for such conditions. Given the importance of early detection and intervention of MH symptoms, a comprehensive examination is essential in evaluating the net benefit of UMHS, particularly for young children.

UMHS for childhood MH problems can manifest in diverse formats and can be deployed across a range of pragmatic settings such as schools, hospital emergency departments (EDs), and in primary care. Over the past decade, the development and evaluation of UMHS for children has increased but has predominantly focused on the school setting (Guo & Jhe, 2021; O’Dea et al., 2021; Wood & McDaniel, 2020). UMHS conducted in schools has several benefits, including the capacity to reach almost all children and mobilizing school resources to support children identified to be at risk of MH problems (Dowdy et al., 2015; Soneson et al., 2020). However, a recent systematic review of schools-based screening identified mixed findings for the feasibility of such approaches (Soneson et al., 2020). Specifically, school staff raised concerns about resource requirements for implementing screening initiatives and whether MH screening was within schools’ remit. On the other hand, parents held highly positive attitudes about school-based screening, finding it acceptable, (Soneson et al., 2020). It is also arguable that parents are best placed to complete the screening instruments and to act on any subsequent recommendations. Firstly, parents typically know their child best, particularly when it comes to emerging social, emotional, and behavioral problems (Teagle, 2002; Tsang et al., 2020). Secondly, where screening identifies risk for MH problems, parents are best placed to facilitate their child’s access to MH services (Teagle, 2002; Reardon et al., 2017; Guo & Jhe, 2021).

The present review had two aims. Firstly, to examine the acceptability and effectiveness of parent reported UMHS initiatives to identify evidence-based best practices and highlight factors that contribute to the successful deployment of UMHS. In this context, ‘acceptability’ referred to the perception among parents that a given UMHS program was agreeable, palatable, or satisfactory (Proctor et al., 2011). ‘Effectiveness’ referred to the ability of UMHS as an intervention to achieve the intended outcomes (Andrews, 1999), in this case, to improve early identification and treatment of child MH symptoms (Dowdy et al., 2015). As such, effectiveness was assessed via the uptake of MH referrals and/or services. Assessing effectiveness is imperative to gauge the accuracy of UMHS initiatives in identifying at-risk children and facilitating referrals to appropriate services only when needed. Secondly, the review aimed to explore the perceived barriers and enablers of UMHS as reported by parents to identify factors that may facilitate parents' continued access and uptake of UMHS initiatives. ‘Barriers’ and ‘enablers’ referred to any economic, personal, or implementation factor that inhibits or facilitates access (Ocloo et al., 2021) to UMHS. By examining the perceived enablers, barriers, and any unintended negative effects associated with UMHS, this review aims to improve knowledge of the factors that contribute to parental engagement with UMHS. A systematic review of this evidence aimed to contribute to a comprehensive understanding of the factors that enhance the implementation and uptake of UMHS, and to identify evidence-based strategies to inform the development of future UMHS protocols that are based on effective, acceptable approaches to suit the diverse needs of stakeholders. This knowledge is vital for the successful implementation and sustainability of UMHS initiatives, which may be important mechanisms for early identification of child MH problems.

## Methods

This systematic review was registered with the International Prospective Register of Systematic Reviews (PROSPERO; https://www.crd.york.ac.uk/prospero; registration number: CRD42022299560). Reporting was guided by the standards of the Preferred Reporting Items for Systematic Review and Meta-Analysis (PRISMA) Statement.

### Inclusion and Exclusion Criteria

Studies that examined UMHS programs in children and adolescents aged from birth to 18 years, and whose aim was to detect mental health symptoms through parent-report were included. We did not restrict study design, although excluded reviews, case studies, commentaries, and conference proceedings. Studies were excluded if the mean age of the child exceeded 18 years; where no parent report was included; the primary focus of was academic outcomes, physical and/or metabolic health; or UMHS was implemented in targeted, clinical, or populations experiencing an acute mental or physical health emergency. Studies evaluating psychometric properties of UMHS measures were excluded. Only studies published in English and in peer-reviewed journals were included, with grey and unpublished literature excluded.

### Search Strategy

We searched the following electronic databases in February 2022: Medline, PsycINFO, EMBASE, CINAHL, Scopus, and Web of Science. A librarian liaison was consulted regarding search term development and database selection. Keywords were developed to cover three domains: 1) “child”, “youth”, “adolescent”, “parent”, or “family”, 2) “mental health”, and 3) “universal screening”, “mass screening”, or “screening”. No restrictions were placed on the start or end date for inclusion. A secondary search was performed by hand-searching reference lists of key publications that emerged during the review process.

### Study Selection, Data Extraction, and Quality Assessment

One author (S.B.) reviewed all titles and abstracts. A second, (J.N.) independently reviewed a random sample (20%). One author (S.B.) reviewed all full-text articles. A random sample (20%) was evenly distributed across four authors (T.C., L.T., C.C., and R.M.). Data related to study design, aims, sample characteristics, demographics, screening protocols, acceptability, effectiveness, enablers, and barriers was extracted. Study quality was evaluated using the Mixed Methods Appraisal Tool (MMAT; Pace et al., 2012). The MMAT required two reviewers to independently appraise each article, which were evenly divided between five authors (S.B., L.T., C.C., R.M., and T.C.). Studies were included regardless of quality.

## Results

**Fig. 1:**
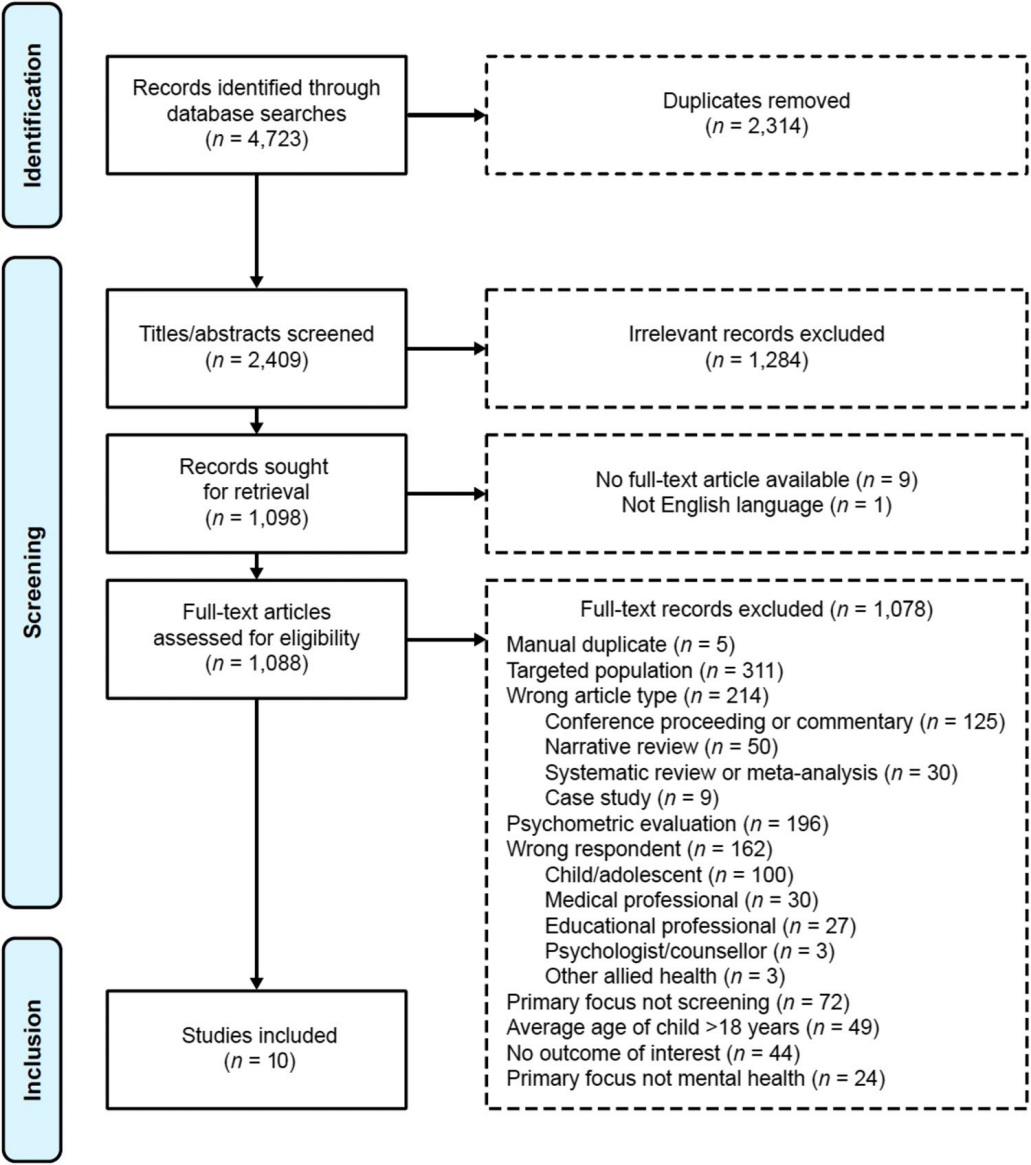
PRISMA Flow Chart for the Articles Included in the Systematic Review

A total of 4723 articles were identified and of these, ten studies met inclusion criteria (see Fig. 1 for PRISMA flow chart).

### PRISMA Flow Chart for the Articles Included in the Systematic Review

A comprehensive overview of the study characteristics is available in Table 1. Studies were predominantly quantitative in design (*n* = 6) and conducted in the United States (*n* = 6). Three settings emerged, spread equally between Pediatric Well Child Visits (PWCVs) (*n* = 4), schools (*n* = 3), and hospital Emergency Departments (EDs) (*n* = 3). Samples ranged from 19 to 1555 parents, the majority of whom, where reported (*n* = 5), were almost exclusively female. However, where reported (*n* = 5), child gender was evenly distributed (48.0 – 55.0% female). Where child age was reported (*n* = 7), three studies focused on children under 11 years, three on adolescents aged 11 – 18 years, and one on children and adolescents between 4 – 18 years. Where reported, the primary respondent was a parent and/or caregiver (*n* = 7), followed by a parent–child dyad (*n* = 2), and the child (*n* = 1). Response rates were reported in most studies (*n* = 8), with significant variability between studies. At the lower end, as few as 15% of eligible participants responded to the invitation to participate in a semi-structured interview conducted by Childs-Fegredo and colleagues (2021). Furthermore, of those parents who responded to Soneson and colleagues’ (2018) open-ended ‘Statements’ (*N* = 290), response rates varied from 29% (*n* = 83; Statement 5: Potential Harms of Screening) to 44% (*n* = 128; Statement 4: Potential Benefits of Screening). Elsewhere, response rates of eligible parents ranged from one-third (Berger-Jenkins et al., 2012), 41% (Jonovich & Alpert-Gillis, 2014), 57% (Pailler et al., 2009), 70% (Fothergill et al., 2013), and 91% (O’Mara et al., 2012), respectively.

**Table 1:** Description of Included Studies

Author, year	Country	Study Design	Sample Characteristics						Screening Characteristics							
			*Parent* *(n)*	*Child* *(n)*	*Child* *(% female)*	*Parent* *(% female)*	*Child age range (M)*		*Setting*		*Proband(s)*		*Response Rate(s)*		*Mental Health Domain(s)*	*Instrument(s)*
Berger-Jenkins et al., (2012)	United States of America	Quantitative Non-Randomized Chart Review	NR	*N* = 114	44.3%	NR	5 – 12 years (*M* = 7.8)		Well-child Visit		Parent/ Caregiver		1/3rd (*N* = 114) of eligible parents completed surveillance instrument 82.5% (*N* = 94) went on to complete the PSC-17. Reason for non-completion not documented.		‘General’ Child MH	Pediatric Symptom Checklist (PSC-17)
Childs-Fegredo et al., (2021)	United Kingdom (England)	Qualitative Semi-Structured Interview	*N* = 19	N/A	NR	89.0%	3 – 11 years (NR)		School		Parent/ Caregiver		*N* = 122 approached, 24 responded, 20 agreed, 19 completed. Reason for non-completion not documented.		NR	n/a
Fothergill et al., (2013)	United States of America	Quantitative Exit Survey and Qualitative Interview	*N* = 120 *n* = 24^b^	*N* = 120	49.0%	87.0%	4 – 10 years (*M* = 7.0)		Well-child Visit		Parent/ Caregiver		*N* = 172 eligible, *N* = 120 (70%) completed screener Of those ineligible (*n* = 52), 12 (23%) declined, 40 for ‘various reasons’, e.g. Clinics concerned about workflow (n = 14), or technical difficulties (*n* = 8).^c^		General Health Risk; Anxiety; Parental Depression; ‘General’ Child MH	SCARED-5, PSC-17, Strengths and Difficulties Questionnaire (SDQ)
Jonovich and Alpert-Gillis (2014)	United States of America	Quantitative Non-Randomized Chart Review with Control Comparison	*N* = 292	N/A	55.0%	NR	11 years		Well-child Visit		Parent/ Caregiver		*N* = 664 children attended during screening period. 41% (*N* = 272) of those eligible completed the screener ‘Every other child’ (*N* = 146) was included. Comparison group (N = 146) was taken from every third child.		‘General’ Child MH	PSC-35
Moore et al., (2022)	United States of America	Prospective Cohort Study	*N* = 330	N/A	NR	73.3%	NR		School		Parent/ Caregiver		NR		‘General’ Child MH	BASC-3 BESS, PSC-17
O'Mara et al., (2012)	United States of America	Quantitative Descriptive	*N* = 300	*N* = 294	48.0%	72.0%	NR (*M* = 15.2)		Hospital ED		Parent–Child Dyad		*N* = 451 presented to ED. *N* = 125 excluded due to medical severity, English language, sedation, cognitive impairment. Of eligible (*N* = 336), 91% (*N* = 305) parents and 89% of children (*N* = 299) consented. N = 5 dyads excluded (presented for suicide attempt).		Suicide Risk	Mental Health Check-up in the Emergency Department Adolescent/ Parent Questionnaire
Pailler et al., (2009)	United States of America	Qualitative	*N* = 59	*N* = 60	51.6%	73.0%	12 – 18 years (*M* = 14.1)		Hospital ED		Parent–Child Dyad		Eligible if child aged between 12 – 18 years and accompanied by a parent. Of the* N* = 115 identified by researchers,* N* = 9 ineligible. 57% (*N* = 60) consented. 1 parent withdrew. Reason for non-completion & withdrawal unknown.		Depression	n/a
Soneson et al., (2018)	United Kingdom (England)	Quantitative Descriptive	*N* = 290	N/A	NR	80.3%	NR		School		Parent/ Caregiver		*N* = 290 consented. 44% (*N* = 128) responded to ‘benefits’ (Statement 4), 29% (*N* = 83) to ‘harms’ (Statement 5), and 21% (*N* = 62) ‘general comments’ Reason for non-completion unknown.		NR	n/a
Valla et al., (2019)	Norway	Quantitative	*N* = 1555	N/A	NR	NR	2 years		Well-child Visit		Parent/ Caregiver		Number of eligible parents approached unknown*. N* = 965 parents voluntarily completed survey. At post, N = 675 parents completed survey and ASQ satisfaction items.		Behavioral and Social Development	Ages and Stages Questionnaire (ASQ-II)
Williams et al., (2011)	United States of America	Quantitative	*N = 384*	*N* = 234	NR	NR	NR (*M* = 10.5)		Hospital ED		Child		NR		'General’ Child MH	DISC Predictive Scales

*NR* = not reported

^a^ where studies report pre- and post-screening data, table lists pre-screening data only

^b^where *n* reported this indicates that children and/or parents were part of a larger sample

^c^all reasons not reported

Considerable heterogeneity existed in the UMHS focus areas and instruments used. Across the studies that deployed a UMHS protocol prior to data collection (*n* = 7), the majority (*n* = 5) screened for ‘general’ psychosocial concerns, predominantly (*n* = 4) using a version of the Pediatric Symptom Checklist (PSC). Beyond ‘general’ MH, screening trends appeared related to setting. For instance, PWCVs focused on child development (*n* = 1) and parental depression (*n* = 1) screening, whereas EDs focused on child depression (*n* = 2), suicide and psychosocial risk factors, such as alcohol use, eating disorders, and anxiety (*n* = 1), and schools focused on behavioral and emotional wellbeing (*n* = 1). Given the limited number of studies in each setting, caution regarding the interpretation of these trends is necessary.

### Acceptability

Seven studies explored the perceived acceptability of UMHS by parents. Two components of acceptability emerged: (1) perceived ‘appropriateness’ or ‘helpfulness’, and (2) perceived satisfaction. The latter encompassed parents’ experiences with the screening device, their comfort during screening, and the likelihood of recommending the screening to others or participating in future. Parents in four studies completed screening prior to measuring perceived acceptability (Fothergill et al., 2013; Moore et al., 2022; Valla et al., 2019; Williams et al., 2011).

#### Appropriateness and Helpfulness

Five studies assessed the perceived appropriateness or helpfulness of UMHS (Table 2). Across all settings, most parents regarded UMHS as appropriate (Moore et al., 2022; O’Mara et al., 2012), were supportive of UMHS (Pailler et al., 2009; Soneson et al., 2018), or felt that it was helpful for their child (Williams et al., 2011). Where reported, there were no significant differences in the perceived helpfulness of UMHS based on receiving a positive or negative screening outcome, although significant personal and cultural factors were detected, with African American parents and those with a prior history of MH issues more likely to perceive UMHS as useful (Williams et al., 2011). Gender differences emerged in one study (O’Mara et al., 2012), with mothers (73% of participating caregivers) rating all domains as more important to screen for compared to fathers, except suicide risk, which was considered equally important.

**Table 2:** The Perceived Acceptability of UMHS by Parents

Author (year)	Setting	Sample Size (Parent)	Assessment type	Summary of Key Finding
O’Mara et al., (2012)	Hospital ED	*N* = 300	Quantitative^a^	The majority regarded the ED as appropriate to screen for suicide risk (92%), alcohol abuse (91%), depression (90%), eating disorders (90%), dating violence (88%), anxiety (87%), and behavior problems (84%) Half (53%) were willing to participate in UMHS in the future.
Pailler et al., (2009)	Hospital ED	*N* = 59	Qualitative^a^	90% ‘supported’ UMHS for their child in the ED.
Williams et al., (2011)	Hospital ED	*N* = 384^d^	Quantitative ^a,b^	The majority rated screening as ‘highly acceptable’ (82%) and ‘helpful’ (65.1%), and were ‘not distressed’ (97.7%) by the screener. The majority trusted the security (89%), and privacy (87%), and felt the length was ‘just right’ (88%), and ‘not boring’ (88%). The majority (92.9%) would recommend screening to others.
Fothergill et al., (2013)	Well-child Visit	*N* = 120^d^	Mixed*^a^	Most felt UMHS was a useful way to ask routine questions (95%) and share concerns (89%). Only a minority felt that screening too long (4%), or that the electronic screening device was difficult to use (7%)
Valla et al., (2019)	Well-child Visit	*N* = 1555^d^	Quantitative^s^	The majority were comfortable answering screening questions about their child’s MH (95%), about the concerns they raised (91%), about disruptions experienced due to their child’s difficulties (87%) and would recommend screening to others (95.6%). 79% felt screening should be made available to all parents at their visits
Moore et al., (2022)	School (Pre-K – Grade 1)	*N* = 330^d^	Quantitative^a^	Strong agreement that screening was appropriate, and that school was an important setting to identify at-risk children. 92.5% would participate in future
Soneson et al., (2018)	School (K – 6)	*N* = 290	Qualitative	82% ‘supported’ UMHS for their child in the school setting, and 85% would participate again in future

^a^authors collected data pertaining to the perceived appropriateness or helpfulness of UMHS

^b^authors collected data pertaining to the perceived satisfaction of UMHS

^c^Quantitative measures were used to assess all parents, with a qualitative interview for a random subsample (20%)

^d^Parents in these studies completed screening prior to the authors assessing their perceptions of acceptability

#### Satisfaction

Three studies addressed the perceived satisfaction with UMHS (Fothergill et al., 2013; Valla et al., 2019; Williams et al., 2011). As detailed in Table 2, where asked, most parents (88%) indicated that they did not find the screening ‘boring’, and thought the length was just right (Williams et al., 2011). Similarly, only a small minority (4%) felt screening took too long, or that the device was difficult to use (7%) (Fothergill et al., 2013). Most parents also trusted the security (89%) and privacy (87%) of the screening environment, were comfortable answering questions about their child’s MH (95%), did not find the questions ‘intrusive’ (Forthergill et al., 2013), and were not ‘distressed’ (97.7%) by the UMHS received (Williams et al., 2011). Finally, when asked, between 92.9% (Williams et al., 2011) and 95.6% (Valla et al., 2019) would recommend screening to others, and between 85% (Soneson et al., 2018) and 92.5% (Moore et al., 2022) indicated their willingness to participate in future.

### Effectiveness

Two studies examined the effectiveness of UMHS to determine whether it improved the identification, referral, or treatment of MH problems (Berger-Jenkins et al., 2012; Jonovich & Alpert-Gillis, 2014). Both occurred at PWCVs and used a random chart review of medical records for patients seen prior to the introduction of UMHS. Berger-Jenkins and colleagues (2012) reported an eight-fold increase in parental disclosure of MH concerns regarding their five-to-twelve-year-old child post-screening implementation, compared to before. However, this increase did not translate to a change in practitioner’s behavior in inquiring about MH problems. Despite a trend of more clinicians initiating a ‘work-up’ of diagnostic interviews and validated tests after screening, fewer clinicians referred directly to co-located MH services after screening was implemented. In Jonovich and Alpert-Gillis’ (2014) sample of parents of 11–12-year-olds, those who completed screening (*N* = 143) were significantly more likely to be referred to a co-located (2% vs. 18%) or community counselling service (10% vs. 24%) and to attend their co-located counselling service (0.5% vs. 10%), community counselling service (1% vs. 10%), or psychiatrist appointment (0% vs. 3%). Controlling for pre-existing MH diagnoses, screening significantly predicted parents’ referrals to counselling services, appointment attendance, and raising of concerns with the primary care provider (PCP) at their *current* visit (36% vs. 25%). However, screening did not significantly predict future MH diagnosis (25% vs. 21%), the prescription of psychotropic medication by a psychiatrist (2% vs. 0.5%) or PCP (14% vs. 16%) or raising concerns at their *next* visit with a PCP (17% vs. 12%) or social worker (20% vs. 13%). Taken together, these results suggest that UMHS is associated with increased attendance at treatment appointments (Jonovich & Alpert-Gillis, 2014), and discussion of MH concerns, although mixed support regarding referrals (Berger-Jenkins et al., 2012; Jonovich & Alpert-Gillis, 2014).

### Barriers to UMHS

Four studies reported on the barriers and concerns parents raised in relation to UMHS (Table 3), although, crucially, only one (Fothergill et al., 2013) required parents to complete screening prior to assessing their perceived barriers or concerns regarding UMHS. Several themes emerged as a function of setting. Parents in hospital EDs both raised privacy as their greatest concern (O’Mara et al., 2012; Pailler et al., 2009), and a small number were concerned about the stigma of ‘what others might think (O’Mara et al., 2012), or of the appearance of being ‘singled out’ for screening (Pailler et al., 2009). In schools, practical themes emerged pertaining to the burden of responsibility (Childs-Fegredo et al., 2021), and inadequate staff training (Childs-Fegredo et al., 2021; Soneson et al., 2018). Across both settings, a minority were concerned that screening could lead to discomfort (Soneson et al., 2018), distress (O’Mara et al., 2012), false negatives (Soneson et al., 2018), or misinterpretation (O’Mara et al., 2012; Soneson et al., 2018).

**Table 3:** The Perceived Barriers and Benefits/Enablers of UMHS by Parents

Author (year)	Setting	Assessment type	Sample Size (Parent)	Perceived Barriers	Perceived Benefits/Enablers
O’Mara et al., (2012)	Hospital ED	Quantitative Survey	*N* = 300	Parents ‘agreed’ to ‘strongly agreed’ that screening raised privacy concerns (33%), that screening could distress their child (26%), be time consuming (21%), unnecessary (14.5%), or stigmatizing (7%)	NR
Pailler et al., (2009)	Hospital ED	Qualitative	*N* = 59	Parents identified a discomfort with disclosing sensitive information, the fear of stigma, and the appearance of being ‘singled out’ for screening	Parents were conditionally supportive of ED screening if parents were involved in the screening and were included in the sharing of the results
Fothergill et al., (2013)	Well-child Visit	Mixed^a^	*N* = 120^b^	NR	A subsample (20%) qualitatively identified that UMHS deployed as a pre-screen prior to their visit streamlined conversations with their provider and the increased efficiency of the visit
Childs-Fegredo et al., (2021)	School (K – 6)	Qualitative	*N* = 19	A lack of time and resources; potential stigma; concerns around accuracy and reliability; the burden of staff responsibility and training required	Some parents believed that early identification programs could foster deeper relationships between parents and school staff; enhance MH literacy; and reduce stigma
Soneson et al. (2018)	School (K – 6)	Qualitative	*N* = 290	A minority were concerned about false negatives (7.7%); that emotional difficulties are hard to detect in children (6.2%); the potential for screening to be harmful without adequate staff training (5%); result misinterpretation (5%); or the risk of discomfort for children (4%)	Some parents perceived UMHS as beneficial for their child (27%), by facilitating timely help-seeking (22%), reducing self-harm and suicide by offering guidance and support (9%), and prevent children at-risk from ‘slipping through the net’ (3%)

*NR* = Not reported

^a^Quantitative measures were used to assess all parents, with a qualitative interview for a random subsample (20%)

^b^Parents in these studies completed screening prior to the authors assessing their perceptions of acceptability

### Benefits or Enablers of UMHS

Four studies reported on the perceived enablers of UMHS (Table 3), with only one (Fothergill et al., 2013) requiring parents to complete screening prior to assessing their perceptions of UMHS. In school settings, parents highlighted the potential for UMHS to facilitate timely help-seeking (Soneson et al., 2018), increase MH literacy, reduce stigma, and foster relationships between parents and school staff (Childs-Fegredo et al., 2021). When deployed as a pre-screener prior to a well-child visit (Fothergill et al., 2013), parents felt that UMHS enhanced the efficiency of their visit by saving time and focusing the conversation with their PCP. Parents in the ED were conditionally supportive of UMHS, given there was parental involvement in the screening and in the sharing of results (Pailler et al., 2009).

### Quality Assessment

Study quality was evaluated using the Mixed Methods Appraisal Tool (MMAT; Pace et al., 2012). The two qualitative studies (Childs-Fegredo et al., 2021; Pailler et al., 2009) were high quality, meeting all seven criteria. Of the three non-randomized quantitative studies, one met all criteria (Jonovich & Alpert-Gillis, 2014), one exhibited moderate quality (five of seven) due to inappropriate measures and insufficient data to determine sample representativeness (Valla et al., 2019), and one was moderate-to-low quality (four of seven), with issues related to sample representativeness, confounder control, and insufficient data to determine the completeness of the outcome data (Berger-Jenkins et al., 2012). Three quantitative descriptive studies demonstrated moderate quality, meeting five criteria. Two exhibited non-response bias and insufficient use of validated measures (Moore et al., 2022; Williams et al., 2011), and/or insufficient sample representativeness (Moore et al., 2022; O’Mara et al., 2012). The two mixed-methods studies were high quality (six of seven criteria), due to insufficient sample representativeness (Fothergill et al., 2013; Soneson et al., 2018).

## Discussion

This systematic review summarized the published literature on the acceptability and effectiveness of parent reported UMHS for children from birth to 18 years, and identified barriers and enablers that may improve parental engagement. Only ten articles were identified, indicating a significant research gap, despite the important role parents play in the identification of child mental health (MH) concerns (Reardon et al., 2017). Generally, parents viewed UMHS as acceptable, highlighting the perceived benefits in early MH symptom identification and enhanced communication with healthcare providers (HCPs) and school staff. The limited evidence on effectiveness showed that UMHS led to appropriate referrals to MH services, improved appointment attendance, and facilitated discussions around MH concerns with HCPs. Barriers included privacy concerns, stigmatization, and concerns about screening accuracy.

Our first goal was to consider whether parent reported UMHS is worthwhile to by examining its acceptability and effectiveness. Acceptability was the most promising factor evaluated, and a central focus existing research. Across all study settings, parents generally perceived UMHS as appropriate and helpful (Moore et al., 2022; O’Mara et al., 2012; Soneson et al., 2018; Williams et al., 2011). This is crucial, as for UMHS to be effective, it must first be acceptable to parents, motivating their engagement in future initiatives (Moore et al., 2022). Our findings align with the high acceptability reported by other key stakeholders, including school principals (Woods & Ellis, 2022), school counsellors (O’Dea et al., 2021), adolescents (Robinson et al., 2011; Zuckerbrot et al., 2007), and HCPs (Pailler et al., 2009; Williams et al., 2011; Zuckerbrot et al., 2007), without whose support, the successful implementation of UMHS would not be possible. However, while we can say broadly that parents found UMHS to be acceptable and worthwhile, the reliability of this conclusion is limited by the diverse methodologies used to assess acceptability across the included studies.

The effectiveness of parent reported UMHS was less frequently documented, with only two studies including data related to the identification, management, referral, or treatment of MH problems (Berger-Jenkins et al., 2012; Jonovich & Alpert-Gillis, 2014). Both studies found that screening increased parental disclosure of MH concerns to HCPs post-screening. This is significant, as parental help-seeking is a key determinant for the timely treatment of child MH issues (Hacker et al., 2006). Despite the potential for increased parental disclosure, evidence supporting increased referrals to treatment following UMHS was limited. One study found UMHS referrals and improved attendance (Jonovich & Alpert-Gillis, 2014) whereas the other found fewer clinician referred to MH services after screening (Berger-Jenkins et al., 2012). These findings highlight the importance of further research to assess whether UMHS increases parental disclosure leading to appropriate referral and treatment, and ultimately improving outcomes for children with MH concerns.

Our second goal was to explore the practical implications of UMHS by investigating parents’ perceptions of the barriers and enablers affecting their uptake of UMHS. The most frequently cited concerns were about privacy and confidentiality (Fothergill et al., 2013; O’Mara et al., 2012; Pailler et al., 2009), accuracy and reliability (Childs-Fegredo et al., 2021; Soneson et al., 2018), and stigma (Childs-Fegredo et al., 2021; Soneson et al., 2018). Interestingly, parents in one study (Childs-Fegredo et al., 2021) noted that UMHS could both increase stigma by ‘singling out’ at-risk children, *and* reduce it, by enhancing MH literacy. Despite these barriers, three quarters of parents in Fothergill and colleagues’ (2013) study were satisfied with the privacy and confidentiality of the screening received, highlighting the complexity of parents’ attitudes toward UMHS. Educating parents about the aims and processes of UMHS, along with assurances about privacy, could help alleviate these concerns, especially since previous findings suggest that parents often feel uninformed about the screening process (Wissow et al., 2013). The lack of quantitative data and studies comparing parent perceptions of UMHS from pre- to post-screening makes it difficult to form conclusions about the impact of these barriers and their influence on UMHS uptake. However, overall, few parents reported barriers, and most did not believe UMHS would be harmful to their child.

Parents identified several perceived benefits or enablers of UMHS. Specifically, they believed that UMHS would facilitate timely help-seeking (Soneson et al., 2018) and improve communication with key stakeholders involved in their child’s wellbeing, such as school staff (Childs-Fegredo et al., 2021), and HCPs (Fothergill et al., 2013). Additionally, parents felt that the universal delivery of UMHS could reduce stigmatization (Childs-Fegredo et al., 2021; Pailler et al., 2009). Notably, much of the research relies on respondents’ self-reported views, which, though valuable, can be subject to bias. To date, no study has systematically examined the benefits and enablers following parent completion of UMHS. Understanding these factors is crucial for prioritizing elements that facilitate UMHS uptake and improve its effectiveness, such as referral and help-seeking rates for child MH concerns (Burns & Rapee, 2021). Given the overall scarcity of data, caution is necessary when drawing conclusions about the relative importance of barriers and enablers and their impact on help-seeking and service uptake. Future research on these factors is essential to assess the acceptability and effectiveness of implementing UMHS as a population-based approach.

### Limitations and Recommendations for Future Research

Despite the findings in this review suggesting that UMHS is acceptable, with few barriers and a number of enablers, several methodological limitations must be taken into account when interpreting the conclusions. We describe these limitations and offer focused recommendations for future research in the field.

#### Limitation 1: Methodological Variability and Reporting Inconsistencies in UMHS Studies

This review identified significant variability in methodology across studies evaluating the acceptability and effectiveness of UMHS, including diverse terminology to capture key constructs, various screening instruments with different levels of psychometric rigor, and inconsistent reporting of important methodological processes across included studies, such as response and attrition rates. More specifically, key constructs such as acceptability were operationalized inconsistently, with only limited attention to crucial aspects for promoting screening uptake. For instance, the perceived convenience of UMHS, such as the usability and time requirement are key to its adoption (Moore et al., 2022; Proctor et al., 2011), but only one study directly explored parents’ experiences of using an electronic UMHS device (Fothergill et al., 2013), and only two examined the perceived time burden when completing screening (Fothergill et al., 2013; Williams et al., 2011). Only two studies directly asked parents about privacy concerns (Fothergill et al., 2013; O’Mara et al., 2012) or their or the child’s level of distress during screening (Moore et al., 2022; O’Mara et al., 2012). Yet, privacy, time requirements, and potential distress were reported elsewhere by parents as potential barriers to UMHS acceptance (Childs-Fegredo et al., 2021; Pailler et al., 2009; Soneson et al., 2018). Further, no study examined potential unintended negative effects of parent reported UMHS, which is vital to considering the concerns of policymakers and providers that UMHS initiatives may inadvertently raise parent anxiety or drive unnecessary help-seeking (Jureidini & Raven, 2012), thereby straining already overburdened MH services.

Many studies also used different screening instruments with little rationale for their selection, capturing varied MH domains and hindering the ability to draw meaningful conclusions regarding the acceptability of UMHS. Other methodological inconsistencies were apparent in the reporting of response rates. Two of the ten studies did not report response rates, and for those that did, response rates varied widely from 15 to 91%. In addition, only two of the ten studies reported reasons for screening refusal or non-completion. Finally, very few studies required parents to complete screening prior to researchers examining their perceptions. This lack of direct experience with UMHS is problematic, as their feedback may not wholly reflect the practical challenges, acceptability, or enablers of the screening process.

Future research should aim for more comprehensive reporting of methodological procedures. Specifically, studies should include the specific rationale for selecting UMHS instruments, strategies for standardizing screening processes, privacy protection measures, and documentation of parental refusal, non-completion, or attrition, all factors impacting the acceptance and uptake of UMHS by parents. Reporting on response rates in studies of UMHS is important since low response rates may themselves indicate a lack of parent acceptability of UMHS, warranting further investigation. Moreover, systematic examination of unintended negative effects, such as parental emotional distress, stigma, or rates of false positive or misidentification are essential to alleviate stakeholder concerns and build support UMHS at the individual and site-level to promote an evidence-informed approach to implementation. Adopting standardized terminology for key constructs, such as acceptability, effectiveness, barriers, and enablers, is also essential to ensure the rigorous evaluation of future UMHS initiatives. Finally, it is critical for future research to ensure that parents have undertaken UMHS prior to reporting on their perceptions. Taken together, future studies can improve the comparability, transparency, and reproducibility of UMHS by utilizing consistent terminology and clearly articulating key methodological processes. Doing so may enhance our understanding of UMHS acceptability, better identify and address parental concerns that could impact uptake and advance the implementation of UMHS to provide more equitable and effective initiatives across settings.

To address the inconsistencies in the UMHS instruments selected across the included studies in this review, future studies should aim to standardize a common measure and prioritize brief, psychometrically validated instruments that can be used across different ages and are adaptable to the diverse pragmatic settings in which UMHS initiatives are implemented. For instance, the Pediatric Symptom Checklist-17 (PSC-17) and its associated age-appropriate measures (Murphy et al., 1996). is brief, free, and widely validated, with robust psychometric properties, designed to assess psychosocial symptoms in children across developmental stages. The PSC-17 is validated for children aged four years and older (Gardner et al., 1999). Additionally, companion versions are tailored to infancy (0 – 24 months; Baby PSC; Sheldrick, et al., 2012a, b), and early childhood (18 – 60 months; Preschool PSC; Sheldrick, et al., 2012a, 2012b). These instruments have demonstrated reliability and validity across diverse cultural, clinical, and community samples (Murphy et al., 2016; Stoppelbein et al., 2012). It is crucial for UMHS to adopt such valid, developmentally appropriate instruments to facilitate meaningful comparisons across studies and evaluate child MH outcomes across the developmental trajectory. Doing so would reduce variability in the administration and interpretation of screening instruments, ensuring more accurate and reliable conclusions about the acceptability and effectiveness of UMHS as a population-based strategy.

#### Limitation 2: Poor Representativeness of Diverse Participants

It must also be acknowledged that there was a significant overrepresentation of Western, Educated, Industrialized, Rich, and Democratic (WEIRD) samples in included studies. Given that UMHS is intended as a coordinated, population-level public health approach (Burns & Rapee, 2021; Humphrey & Wigelsworth, 2016), further examination of UMHS in diverse samples is essential to ensure that the implementation of UMHS is inclusive, representative, and thus, truly universal. Further, the focus of this review was parent reported population-level screening, so excluded studies conducted in targeted settings, with at-risk samples such as those in juvenile justice, out-of-home care, medical inpatient facilities, or with specific, previously diagnosed disorders, like Autism Spectrum Disorder. Understanding the complexities of these settings, which often include children who are disproportionally impacted by MH concerns (Childs-Fegredo et al., 2021) is vital to optimizing the use of limited resources to tailor MH screening to the unique challenges and specific needs of vulnerable groups.

Future research should therefore aim to explore the validity, cultural sensitivity and acceptability of parent reported UMHS in culturally diverse populations. For instance, qualitative studies can investigate how diverse cultural beliefs, practices, and conceptualizations of MH influence parents’ perceptions of and engagement with UMHS. Incorporating community-led research, including focus groups, within specific cultural groups or communities, can help ensure that future initiatives are culturally sensitive, relevant, and effective in addressing the unique MH needs of diverse populations and complement existing referral and service pathways.

Another avenue for reaching diverse populations and maximizing access to UMHS is the development of an online UMHS platform. An online platform, offering automated feedback and links to recommended services, could leverage the growth of evidence-based online MH services, making UMHS more accessible and improving uptake, including for rural and remote populations. It could further integrate into various pragmatic settings, preventing unnecessary duplication of screening efforts and reduce resource needs for training, assessment, and follow-up; an essential consideration as many UMHS initiatives are not sustained beyond their initial funding period (Moore et al., 2022). For example, as suggested by Fothergill and colleagues (2013), employing an online screener as a pre-visit tool may streamline discussions between parents and HCPs, a valuable approach when considering that PWCVs typically last only 20 min and must address a broad range of psychosocial and developmental topics. Moreover, online screeners offer the advantage of immediate administration and scoring, allowing parents to concentrate on the screening results and discuss the screening outcome in a shared language with their HCP, which parents in found particularly beneficial. By incorporating triage pathways and providing recommendations to evidence-based supports, it could streamline treatment access, easing the burden on schools and HCPs to identify and recommend appropriate services, particularly considering that UMHS is often conducted simultaneously with other screening priorities, such as developmental (Fothergill et al., 2013), somatic and cognitive concerns (Schonwald et al., 2009), and academic outcomes (Moore et al., 2022). However, it would be important to examine the efficacy of any newly developed online UMHS via randomized controlled trials (RCTs).

The included studies were largely descriptive and observational, and none used an RCT design to test efficacy, which limits causal inferences about the relationship between UMHS and subsequent MH service utilization. RCTs are considered the gold standard for evaluating efficacy and are necessary for future studies to evaluate the extent to which parent reported UMHS can accurately identify child MH symptoms, increase timely and appropriate help-seeking, and improve child MH. Ideally, RCTs would include long-term follow-up to examine the durability of the effects of parent reported UMHS on child MH outcomes over time.

## Conclusion

This is the first systematic review to examine the acceptability, effectiveness, barriers, and enablers of parent reported UMHS for children aged birth to 17 years, 11 months. Despite parents being gatekeepers to identifying child MH symptoms and facilitating service utilization, limited research has explored their perspectives on UMHS. The review found parents generally accept UMHS, regardless of the screening instrument utilized, or the setting in which it was deployed. Parent reported UMHS shows promise in increasing help-seeking and connecting families to MH care, potentially reducing or preventing symptom escalation. However, further research is needed on the acceptability, effectiveness, and possible unintended negative effects of UMHS before large-scale implementation. We recommend standardizing UMHS constructs and measures, and suggest future research employ rigorous RCT designs to ensure that UMHS enhances early identification and timely intervention of child MH problems, to ultimately reduce their prevalence.
